# Application of Silicone in Ophthalmology: A Review

**DOI:** 10.3390/ma17143454

**Published:** 2024-07-12

**Authors:** Tamara Mladenovic, Fatima Zivic, Nenad Petrovic, Sasa Njezic, Jelena Pavic, Nikola Kotorcevic, Strahinja Milenkovic, Nenad Grujovic

**Affiliations:** 1 Faculty of Engineering, University of Kragujevac, Sestre Janjic 6, 34000 Kragujevac, Serbia; tamara.mladenovic@uni.kg.ac.rs (T.M.); jelena.pavic@uni.kg.ac.rs (J.P.); kotorcevic@kg.ac.rs (N.K.); strahinja.milenkovic@fink.rs (S.M.); gruja@kg.ac.rs (N.G.); 2 Institute for Information Technologies Kragujevac, University of Kragujevac, Jovana Cvijica bb, 34000 Kragujevac, Serbia; 3 Faculty of Medical Sciences, University of Kragujevac, Svetozara Markovica 69, 34000 Kragujevac, Serbia; nenadpet@yahoo.com; 4 Faculty of Medicine, University of Banja Luka, Save Mrkalja 14, 78000 Banja Luka, Bosnia and Herzegovina; sanjezic@yahoo.com

**Keywords:** siloxane elastomers, silicone intraocular lens (IOL), silicone oil, posterior capsular opacification (PCO), surface modifications, drug delivery, bionic eye

## Abstract

This paper reviews the latest trends and applications of silicone in ophthalmology, especially related to intraocular lenses (IOLs). Silicone, or siloxane elastomer, as a synthetic polymer, has excellent biocompatibility, high chemical inertness, and hydrophobicity, enabling wide biomedical applications. The physicochemical properties of silicone are reviewed. A review of methods for mechanical and in vivo characterization of IOLs is presented as a prospective research area, since there are only a few available technologies, even though these properties are vital to ensure medical safety and suitability for clinical use, especially if long-term function is considered. IOLs represent permanent implants to replace the natural lens or for correcting vision, with the first commercial foldable lens made of silicone. Biological aspects of posterior capsular opacification have been reviewed, including the effects of the implanted silicone IOL. However, certain issues with silicone IOLs are still challenging and some conditions can prevent its application in all patients. The latest trends in nanotechnology solutions have been reviewed. Surface modifications of silicone IOLs are an efficient approach to further improve biocompatibility or to enable drug-eluting function. Different surface modifications, including coatings, can provide long-term treatments for various medical conditions or medical diagnoses through the incorporation of sensory functions. It is essential that IOL optical characteristics remain unchanged in case of drug incorporation and the application of nanoparticles can enable it. However, clinical trials related to these advanced technologies are still missing, thus preventing their clinical applications at this moment.

## 1. Introduction

Silicon (Si) is a chemical element, while silicone is a synthetic polymer that contains many siloxane functional groups. Silicone, or siloxane elastomers, has been widely used in biomedical applications due to its excellent biocompatibility, high chemical inertness, and hydrophobicity [1]. A wide range of applications includes medical implants, different coatings on various medical devices, small devices (catheters, tubes, and drains), aesthetic implants, and ophthalmological implants [1]. Silicones have long been known to prevent the formation of blood clots; hence, they are used to create blood collection tools like silicone-coated needles and syringes [1]. Silicones are also employed in kidney dialysis and cardiovascular silicone membranes and implants due to their hemocompatibility [2]. Silicone elastomers are translucent, flexible, lubricious, and inert. Hence, they are utilized as tubing for artificial urethras (tubular implants), shunts, drains, and catheters [1,2]. Silicone has been used as a dynamic spacer for the implants of joints or for small joints in orthopedic applications [3,4]. Due to their high air permeability, silicone adhesives are employed in scar and keloid therapy, while silicone rubber is used in prosthetics [5]. In reconstructive aesthetic surgeries, silicone is commonly used as an implant for the breasts, scrotum, nose, cheek, chin, and tights [6].

Silicone is used for different implants and devices in ophthalmology, such as in soft contact lenses including drug-eluting lenses [1,7,8,9], intraocular lenses (IOLs) [10], orbital implants [11,12], glaucoma drainage devices [13,14], tube-shunts for reducing intraocular pressure [15], and many small elements in different devices. 

There are excellent silicone-based IOL designs [16] but complications can still occur, for example, due to bacterial adhesion [10,17,18] or unwanted intraocular pressure changes [19]. Silicone oil has been used for years in ophthalmology as one of the best materials but some issues still can occur, such as retinal toxicity [20].

Different modifications of silicone-based materials used for IOLs are studied to provide enhancement of the lens properties, as well as better patient comfort [21,22]. Surface modification can improve IOLs [23,24] or add a drug-loading function [25,26]. Porous orbital implants have been studied and showed better postoperative recovery and lower inflammatory reactions [11,12]. New directions in material development aim to provide functionality and smart behavior, such as closely mimicking tissue structures and shapes [27] and implantable bioelectronics [28] that can perform as biosensors [29]. Additionally, opto-mechanical eye models have been studied for further industrial applications [30]. Advanced nanotechnology solutions have been studied to provide new functionality and improvements in materials [31,32], including nanocarriers and novel drug delivery systems [33].

This paper presents a review of silicone applications in ophthalmology. Physicochemical and mechanical properties of silicone are reviewed including intraocular lenses (IOLs), with the most important aspects in material and implant designs. Uveal and capsular biocompatibility of IOLs is discussed as influences on posterior capsular opacification. Treatments for retinal detachment and retinal restoration are presented, from the aspect of used biomaterials. Silicone-based implants and devices are reviewed with their advantages and disadvantages. Emerging uses for advanced ophthalmology treatments are shown, including new drug delivery systems, retinal prosthesis, smart devices for treatment, diagnostic and control, and material modifications to provide functional properties, including possible future research directions.

## 2. Physicochemical Properties of Silicone

**Figure 1:**
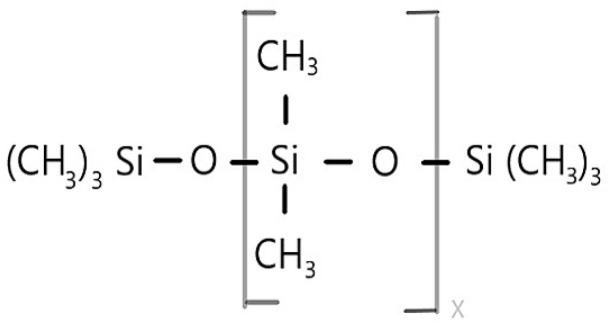
Chemical structure of polysiloxane.

The siloxane backbone, which contains repeating units of silicon-oxygen bonds (-Si-O-Si-O-), is the structural core of polysiloxanes (Figure 1), also known as silicones. Different organic groups are attached to the silicon atoms in the siloxane backbone, showing that silicone is a polymer composed of the inorganic (Si-O) backbone and organic side groups (R) [1,34]. 

Silicon (Si) is electropositive as a chemical element and therefore forms covalent bonds with oxygen. These bonds are called siloxane bonds (Si-O-Si) and form inorganic monomer chains of different lengths with two organic monovalent radicals [1,35,36]. The general formula is -R_2_Si-O, where the substituent R is usually a methyl, ethyl, propyl, phenyl, fluoroalkyl, aminoalkyl, hydroxy, mercapto, hydrogen, or vynil group [35]. For example, silicone atoms attached to a methyl group give polydimethylsiloxane (-Me_2_SiO-), one of the most common forms of polysiloxane polymers [5,37]. Siloxanes have four different functional levels that determine their structure along with the kind of functional groups they possess. Tetrafunctional (Q), trifunctional (T), difunctional (D), or monofunctional (M) monomers or units can exist in different polysiloxane structures, whereas T and Q units form branched and cross-linked molecules that can be observed in elastomers or resins [5,35]. Siloxane polymers can have different structures based on different functionalities of the Si-O unit because the (SiR_2_O)n chain can be adjusted in length or side groups can be introduced or cross-linking can be realized, thus fine-tuning the final properties of the siloxane polymer or silicones [38]. Siloxanes can be synthesized by hydrolysis of chlorosilanes. Intermediates of this reaction are silanols whose hydroxyl groups react to spontaneously form linear or cyclic siloxane oligomers. These are then polymerized by a condensation reaction to form polysiloxanes. This reaction, where two monomers are joined to form a series of repeating units and by-products such as water are removed, is called condensation polymerization. The most common polymer formed by this reaction is the linear polydimethylsiloxane, as previously mentioned [2,38].

Different liquid forms of silicone (emulsions and resin) can further be transformed into an elastomer or adhesive by chemical modifications and reactions. These silicone-based products, or siloxane elastomers, are commonly used in biomedical applications [1]. Since silicon (Si) is an electropositive chemical element, the electronegativity between Si and O is high, resulting in a highly polar covalent Si-O bond. The polarity of the Si-O bond provides high bond energy and good temperature stability. The ionic nature of siloxane bonds gives them significant flexibility [39]. Due to their properties such as high gas permeability, hydrophobicity, and thermal oxidation resistance, they are used in a wide range of applications and usually cannot be replaced by carbon-based polymers [40]. Compared to carbon bonds, the angle of siloxane bonds and the bond distance are large, which contributes to the fact that rotation is not restricted and the rotational energy is very low, making polydimethylsiloxane the most flexible silicone polymer [5,37]. As a result of these chemical characteristics, PDMS degrades at 400 °C but has a relatively low glass transition temperature of −120 °C and is almost always liquid between those two temperatures. The flexibility of the bonds between the polymer chains in PDMS and the presence of methyl side groups provide weak intermolecular interactions between them as well as surface tension [39]. Polysiloxanes are chemically inert, which is supportive of good biocompatibility.

The siloxane backbone-[O-Si(CH3)2]n- determine its inertness and resistance to oxidation and deterioration due to environmental factors such as humidity or temperature change within a wider range [41]. Siloxane chains can freely rotate, thus allowing the orientation of the alkyl groups on the surface. As a result, when the silicone polymer is in contact with the environment, methyl groups predominate on the surface, thus creating a low-energy and chemically inert surface. A very significant phenomenon, the amphiphilic nature of silicone elastomers, can be highlighted, among with their unique properties, and used to provide insight into the adhesive qualities of elastomers when in contact with human skin. Contact phenomena for soft materials are complex, with limited research in wear modeling related to in vivo conditions [42]. Surface tension can be lowered via amphiphilic characteristics. For example, when silicone elastomers are in the air, the contact surface compresses the attached methyl groups, which influences the level of hydrophobicity of the substance. The dipole of the siloxane skeleton dominates in water contacts, thus making the silicone more hydrophobic [35]. Also, some of the advantages of silicone polymers are their high elasticity and good electrical properties; they are optically transparent, low cost, and easy to manufacture, as shown in Table 1. 

Silicone rubber is very durable, with 50–500 years needed to degrade, thus making it a very stable material over time for permanent implants. Silicone can be indefinitely used at 150 °C without affecting any of its properties [1]. However, over time, silicone gradually loses elasticity, with a decrease in strength. Extreme temperature fluctuations (above 200 °C) can induce cracks due to expansion and contraction producing higher stress levels. Higher temperatures increase the chemical reactivity of silicone. For example, a silicone-based lens can perform over 400 million cycles with no changes after >2 years [43].

### 2.1. Silicone Biocompatibility

Biocompatibility, as the most important property, includes the response of the biomaterial to the host in relation to the biomaterial (surface structure, chemical, physical and mechanical properties, and degradability) and those related to the host (age, previous procedures, body mass, drugs taken by the patient, and diseases) [44]. Bioinertness assumes the absence of the genotoxicity or transformation of healthy cells to cancer cells [45,46]. The active interface between the biomaterial and host tissue over time governs the functional behavior of the material, considering that surgical procedures induce inflammatory reactions to some extent immediately after the procedure. Tissue response can be grouped in relation to (1) blood–substance interactions, (2) the discharge of danger signals by damaged cells, (3) an acute inflammation, (4) an ongoing inflammation, and (5) a reaction to a foreign body. Macrophages are among the most critical cells that regulate the biomaterial-host reaction because they play a crucial part in the inflammatory response phases and in tissue repair [45,46]. When a biomaterial interacts with host tissue in the initial phase, a thin layer of blood and plasma proteins will develop on its surface through a process known as the Vroman effect. This protein layer may operate as a barrier, separating the biomaterial from the host tissue and altering the biomaterial properties over time [44,45,46]. In the last stage, where they cause fibrosis, macrophages are most prevalent. By separating the implanted biomaterial from the surrounding tissue, the produced fibrous capsule can impair its performance [45]. Hemocompatibility, as the capacity of the biomaterial to minimize hemolysis and prevent thrombus formation, is also crucial [45].

For the evaluation of biomaterials, a variety of biological factors are significantly important, such as the degree of tissue necrosis and degeneration in the surrounding tissue, cell apoptosis, cell proliferation, endothelization, degree of inflammation, biodegradation, etc. [46]. In a wide range of biomedical applications across all areas of medicine, polydimethylsiloxane serves as a superb illustration of a biomaterial with good biocompatibility [5]. Silicone has a low hemolytic reaction due to its hydrophobicity, which contributes to its good blood compatibility. The chemical makeup, high molecular weight, and relative purity of silicones contribute to their low toxicity. Being nontoxic makes them very suitable for long-term implantations [1]. Due to its hydrophobicity and low surface tension, silicone also has strong hemocompatibility, which lowers the risk of encrustation when in contact with bodily fluids [1]. Due to their chemical and thermal stability, siloxane elastomers are less vulnerable to host tissue attacks and repeated sterilization does not harm them [1]. The excellent biocompatibility of the siloxane elastomers is further facilitated by their physiological inertness, low modulus, antiadhesive characteristics, and good adhesive qualities to specific substrates [5]. 

### 2.2. Mechanical Properties of Intraocular Lenses

Mechanical, optical, and other relevant properties of IOLs (intraocular lenses) and their materials have long been studied [47] following the trend of minimally invasive surgery and the appearance of readily available elastomeric silicone implant materials. In terms of mechanical properties, one of the first considerations was material foldability. Other concerns included chemical purity, surface characteristics, and optical properties. When testing the material for foldability, it should not exhibit creasing, meaning no folding damage and good material recovery. Material strength should also be tested to evaluate resistance to tearing and splitting. Hence, relevant mechanical tests should include tensile testing (resistance to tearing, percentage elongation, and elastic modulus determination), hardness testing, and scanning electron microscopy to evaluate folding behavior [17].

Mechanical characterization of the IOLs is complex since in vivo techniques are essentially non-existent but some of the testing procedures developed for the mechanical testing of the eye contact lenses can be used for lab testing, such as the custom setups that have been used to determine elastic modulus, stress relaxation, and toughness of the contact lenses [48] or optical coherence elastography as a novel proposed method [49]. The three-point bending test of the contact lenses according to ASTM D790 can be used to evaluate stress, relaxation, creep, and elasticity [50]. Some new approaches for in vivo characterization of IOLs have considered optical characterization to monitor its performance [51], implanting microfluidic devices [52] or other advanced biosensors [29] and optical coherence tomography (OCT) techniques for detecting in vivo strain and tissue displacement [53,54].

The tribological or surface properties of IOLs have been investigated by using atomic force microscopy [55]. It was found that different surface roughness profiles can result from manufacturing due to a production technology choice. Surface testing can aid manufacturers in making better choices and achieving optimal characteristics. The characterization of the wear after disposal of lenses is one of the valuable methods to assess wear and geometry changes [56].

To investigate the quality of vision for patients with implanted IOLs, some researchers attempted to create an opto-mechanical eye model for the purpose of simulating psychophysical tests like visual acuity and contrast sensitivity tests, allowing for an easier choice for doctors and patients between different IOL types [30,57].

Cherrier et al. (2010) described two IOL measurement systems, one commercially called OptiSpheric IOL, and the other was a sensor-based approach, commercially called WaveMaste IOL [58]. The important measured parameters are aberration, effective focal length (EFL), power, and modulation transfer function (MTF). They found that the first system was adequate for measuring power, EFL, and MTF according to the EN/ISO 11979 standard, while the second system was good at providing a power map, wavefront, and aberrations of the IOL [58].

Eppig et al. (2014) described an optical setup used for photochromic IOL characterization. Photochromic dyes have been used in the field of IOLs for blue light filtering and similar applications. Authors of the study have measured the kinetics of the photochromic action [59] using a Xenon lamp light source, a filter wheel, and a spectrometer. They did not observe the degradation of the dye under the testing temperature similar to that of the human eye.

## 3. Application of Silicone in Ophthalmology

The human eye is one of the most complex organs of the human body; nevertheless, it was the first organ with a successful transplantation of donor tissue (corneal transplantation) [60]. Even before this transplantation, the first artificial biomaterials were used in the eye [61]. Ophthalmology deals with the anatomy, physiology, diagnosis, and treatment of conditions that affect eye function, such as cataracts, glaucoma, or retinal detachment [60,62]. Many ophthalmological biomaterials have been developed to achieve these objectives. However, polymers have gained the greatest research attention due to their biocompatibility and possibility to make customized flexible materials and are currently the most widely used biomaterials [63]. Their application in ophthalmology includes scleral buckles, corneas, lacrimal ducts, drug delivery systems, glaucoma drainage systems, ocular endotamponades, contact lenses, and intraocular lenses [8,63,64]. Since silicone polymers, or siloxane elastomers, have unique properties and are frequently utilized in ophthalmology, they are used in many of these ocular implants [62,65,66].

### 3.1. Intraocular Lens

**Figure 2:**
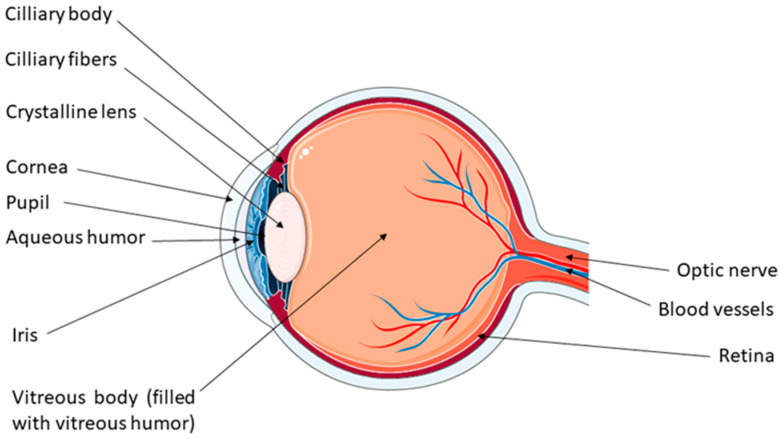
Lens of the eye. Figure modified with line arrows and text after adaptation of “Eye” from Servier Medical Art by Servier, licensed under a Creative Commons Attribution CC-BY 4.0 International License (https://smart.servier.com/smart_image/eye-smart-ov/ accessed on 3 May 2024).

Intraocular lenses are a type of biomaterial used as a permanent implant in the standard procedure to replace the natural lens or they can be implanted over the natural lens in the eye to correct refractive errors that occur because of common vision problems (astigmatism, myopia, and hyperopia) [10,62]. Normal physiology and anatomy of the crystalline lens essentially define the biocompatibility of artificial intraocular lenses, including understanding ophthalmic pathology [67]. The lens is positioned in the eye’s posterior chamber, behind the pupil and iris, as shown in Figure 2. 

In the center of the lens, there is a nucleus surrounded by lens fiber cells that produce the crystalline proteins needed to keep the lens refractive index at 1.42. Fine bands known as zonular fibers connect the ciliary muscles to the lens [68]. The lens of the eye is a transparent biconvex structure and the second part of the eye after the cornea has refractive power. The refractive power of the human eye in its natural non-accommodative state is approximately 15–20 diopters. Specifically, 20% of the refraction is in the lens and 80% in the retina, which has a refractive power of about 43 diopters. The lens enables focusing to different distances to create a sharp clear image of the item on the retina by changing its shape. This process of increasing the dioptric power of the eye is called accommodation and is controlled by the innervation of the ciliary muscle by the autonomic nervous system [67,69,70,71]. When the eye attempts to focus on a near object, the ciliary muscle contracts, causing a drop in tension in the zonular fibers around the lens equator. The increase in curvature of the anterior and posterior lens surfaces, the decrease in lens diameter, and the increase in axial lens thickness contribute to the increase in the dioptric refractive power of the eye [71]. The lens is located behind the iris. It is composed of lipids, sugars, water, low-weight molecules, antioxidants (GSH), and a considerable amount of crystalline proteins. The role of crystalline proteins is to protect the cells of the lens from stress-induced apoptosis, regulate cell growth, act as structural proteins, and, most importantly, maintain the transparency of the lens and its high refractive index [72]. With age, the transparency and refractive power of the lens inevitably decrease, leading to the formation of cataracts. Since there is no other way to treat this condition, the only solution is cataract surgery, i.e., implantation of an artificial lens [62].

### 3.2. Uveal and Capsular Biocompatibility of Intraocular Lenses 

Intraocular lenses must meet a number of requirements, including biocompatibility, the inability to trigger a tissue reaction lasting months, years, or even decades, and good optical qualities for vision restoration [18]. The biological reaction is triggered by the surgical incision, defects of the lens capsule, and disruption of the ocular blood–aqueous barrier once the IOL is implanted [47]. Biocompatibility of intraocular materials can be categorized as either uveal or capsular. Uveal compatibility refers to the foreign body reaction that results from breaking the blood–aqueous barrier following IOL implantation [70,73]. Adsorption of the proteins on the IOL surface starts immediately after the implantation and also facilitates the adsorption of other cells [70,74]. This biological reaction was shown by Jiang et al. [75] in lens epithelial cells from animals that had undergone cataract surgery. The transcriptome of the lens epithelium was significantly altered in 19 genes linked to cataracts at 24 h following cataract surgery. Some of these genes are HMOX1, LCN2, COX-2, CXCL1, CCL2, S100a9, and CSF3/G-CSF, which control the innate immune response and cause chronic inflammation. Extreme inflammatory mediator overexpression starts 1 to 6 h after cataract surgery, peaks at 24 h, and then starts to decline 3 days later. These findings thus demonstrate that proinflammatory cytokines are created right away following surgery [75]. The provision matrix is formulated after the deposition of blood proteins on the surface of the biomaterial. The basis of the provision matrix is fibrin (its precursor is fibrinogen), which initiates the recruitment of fibroblasts and inflammatory cells. An inflammatory response is triggered after the blood clot formation by adsorbed fibrin which attracts phagocytes. Cell adhesion proteins fibronectin and vitronectin also regulate the inflammatory response by initiating the invasion of the IOL surface by monocytes, which then become activated macrophages. Macrophages then fuse and create giant foreign body cells. Thrombin from blood clots also has a role in attracting phagocytes and affecting the regeneration of the damaged host tissue. Platelet-derived growth factor-4 (PF4), platelet-derived growth factor (PDGF), and transforming growth factor-beta (TGF-b) are released from the formed blood clot by activated platelets and function to attract fibroblasts [46]. Activated TGF-b binds to LECs on the cell surface, which in turn triggers SMAD proteins as signaling pathways leading to transcription of the TGF-b gene, Rho GTPases activation, and further stimulation of the PI3/Akt and MAPK pathways [76]. These processes are related to the epithelial–mesenchymal transition (EMT), matrix contraction, myofibroblast formation, cell differentiation, and inhibiting the normal LEC signaling pathways [76]. Inflammatory cellular deposits on the lens surface are common up to a year after cataract surgery. This biological process involves two different types of cells. The first kind of cells are tiny fibroblast-like cells that peak after a month, while the second kind are foreign body giant cells that peak after three months. The large cells degenerate, detach from the surface, and leave an acellular proteinous membrane behind, which typically encloses the IOL, isolating it from the surrounding tissue [70,74].

**Figure 3:**
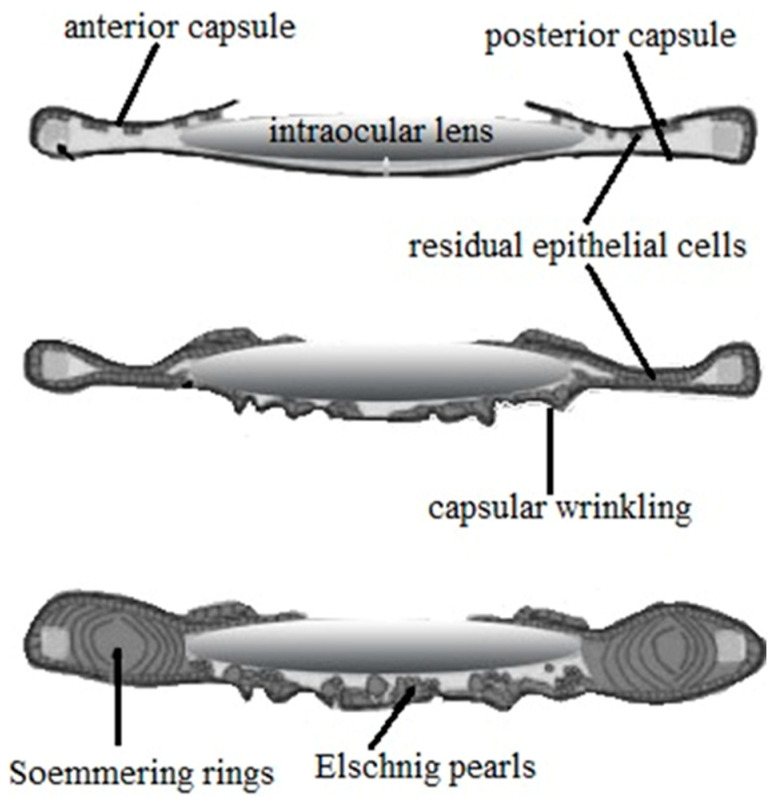
Posterior capsular opacification of the intraocular lens (reprinted from Progress in Retinal and Eye Research, Vol 82, May 2021, 100905, I.M., Wormstone, Y.M., Smith, A.J.O., Eldred, J.A., Posterior capsule opacification: What’s in the bag? [77] Copyright (2021), with permission from Elsevier.

Capsular biocompatibility, or the interaction between the IOL and the remaining epithelial cells of the lens in the capsular bag, is of crucial importance. It is significant to note the two different cell types that make up the epithelium of a normal crystalline lens. The anterior epithelial cells (“A” cells) form a sheet that is continuous with the equatorial cells of the lens (“E” cells). E cells begin to migrate to the posterior lens capsule when they are disturbed by cataract surgery, unlike A cells. Through the epithelial–mesenchymal transition (EMT), A-cells develop into fibroblast phenotypes but do not tend to proliferate. Near the lens equator, E cells, which are nuclei-free and express crystallins (cytoplasmic proteins), increase their size and shape to so-called Soemmering rings and Elschnig pearls (Figure 3) [69,70]. Alpha smooth muscle actin (a-SMA) expression causes these fibrotic cells to proliferate and grow along the posterior area, where they secrete an abnormal extracellular matrix, which is what defines EMT. EMT is significantly regulated by EMC components via autocrine processes, integrin signaling, and growth factor signaling. In particular, EMT has been linked to both fibroblast growth factor (FGF) and transforming growth factor-beta (TGF-b). The opacity of the intraocular lens is the product of both of these transformed cell phenotypes through EMT [69].

### 3.3. Silicone Oil in Retinal Detachment Surgery

For decades, silicone oil has been used as a standard material in eye surgery for long-term internal intraocular endotamponade and has become an essential tool in the treatment of very difficult situations in vitreoretinal surgery [65,66,78,79,80,81].

Silicone oil was first described as an intraocular tamponade for retinal detachment in 1962 by Cibis and colleagues [82]. These authors implanted silicone oil in the corpus vitreum of animals to supply permanent support to the retina. Silicone oil is a synthetic substance composed of repeating units of siloxane (polydimethylsiloxane). Different compositions of silicon oil have been used, with 1500, 2000, or 5000 centistokes [65]. Features of silicone oil that make it a useful tamponade agent include surface tension and viscosity.

**Figure 4:**
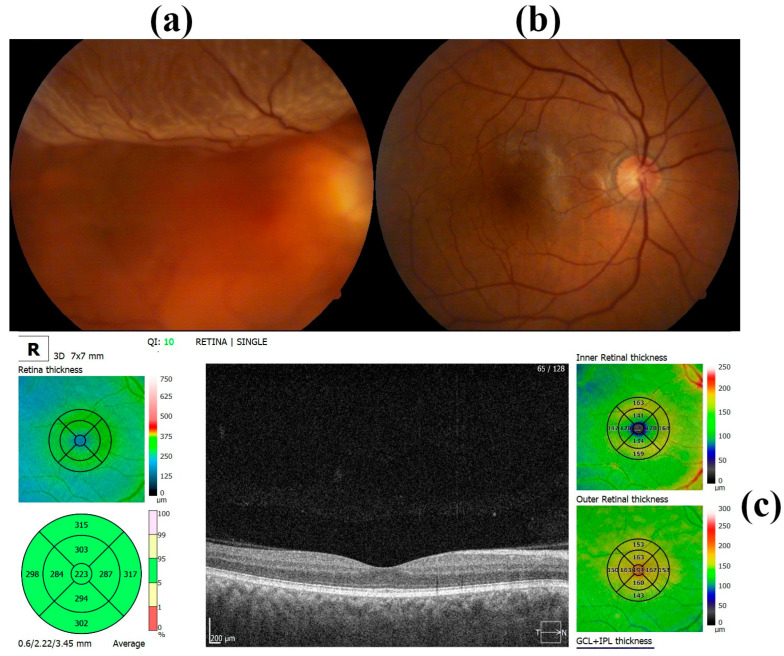
Medical image of (**a**) rhegmatogenous retinal detachment in the right eye, (**b**) same eye after successful pars plana vitrectomy with 5000 cs silicone oil tamponade and anatomical reattachment of the retina, and (**c**) SD-OCT scan of the same eye 4 months after surgery with a normal retinal layer architecture.

Silicone oil has been used as a standard material in complicated retinal detachment [65,66,79,80,81,83] and, recently, as a collapsible capsular vitreous for severe retinal detachment (Figure 4) [84,85]. It has been used to restore retinal detachment that occurred due to proliferative vitreoretinopathy, trauma, or traction [86]. Different compositions of silicone oil have been used, with 1500, 2000, or 5000 centistokes [65]. Due to its cataractogenic effect in phakic eyes as well as the risk of increasing intraocular pressure, silicone oil requires removal after a few months. Some challenges with the long-term presence of silicone oil in the eye still exist [19,20,87] and different approaches have been investigated to overcome those challenges [88,89]. Also, there are still challenges associated with some existing conditions for the use of silicone oil and subsequent effects on patient vision, such as glaucoma [90] or diabetic macular edema [91,92], because some conditions like diabetes have a significant influence on the central corneal thickness [93]. Laser surgeries need to consider silicone oil presence [94,95].

Silicone oil can be easily sterilized and does not show chemical activity [96]. Properties like transparency and oxygen permeability make it suitable for ophthalmological applications, with the possibility to produce different densities, whereas light silicone (approx. 1500 centistokes) can float on water and the dense one sinks (approx. 5000 centistokes) [97]. Silicone oil is used to fill the vitreous cavity when the vitreous body is removed. High surface tension enables the tamponade of the neuroretina. New proliferative vitreoretinopathy is prevented due to limiting the motion of proliferative cells and other mediators, including the presence of blood and fibrin, since silicone oil does not mix with water [98]. There are no effects from the atmospheric pressure that enable patients to freely travel [98]. Possible emulsification of the silicone oil can occur and is associated with negative consequences but higher viscosity is less prone to it [99]. Hence, silicone oil is used as a temporary implant, usually 3–6 months [100].

### 3.4. Silicone-Based Materials for Implants and Devices

Silicone-based implants were developed to improve vision, regulate intraocular pressure, and solve problems with the retina. Over the past 70 years, there has been a significant advancement in the material and design of intraocular lenses. Nowadays, materials used for IOLs are silicone, acrylic (hydrophobic and hydrophilic acrylic), collamer, polycarbonate, and poly (hydroxyethyl methacrylate) (PHEMA) copolymer hydrogel [101]. Different blends are studied, such as the collamer blend (60% of HEMA, 36% water, 3.8% benzophenone, and 0.2% collagen) that enables hydrophilicity and the exchange of gases and nutrients with the eye anterior chamber.

The original intraocular lens biomaterial, polymethyl methacrylate (PMMA), was created in 1949 and was the only implantable for more than three decades. It was constructed of hard plastic and, due to its rigidity, it required a surgical procedure with a significant incision. Astigmatism developed after surgery as a result. Charles Kelman developed the phacoemulsification surgical procedure in the 1970s, which made it possible to minimize the size of the incision [22]. This created the opportunity for the development of new ocular biomaterials, such as silicone foldable IOLs, which improved the safety and effectiveness of cataract surgery [22,102]. 

Silicone was used to create the first commercial foldable intraocular lens [62]. Additionally, silicone IOLs are flexible and made to be implanted with a small incision [68]. The optical diameter of silicone intraocular lenses (IOLs) ranges from 5.5 to 5.6 mm and their refractive index ranges from 1.41 to 1.46 [10]. However, for a single-piece foldable lens with 2 open loops, the optic diameter is 6.0 mm, though injector systems can make the incision even smaller (2.2 to 2.4 mm) [73]. Silicone hydrogels are often used as IOLs because they can easily be folded and unfolded intraoperatively [71]. Single-piece (all-one material) and multi-piece (optic and haptic parts are made from different materials) intraocular lenses can be used. The optic part gives the lens refractive function and the haptic part attaches the lens to the structures surrounding the intraocular lens [62]. Special lens designs have also been developed, including fluid-filled lenses, open-bag lenses, and modular lenses. Depending on the location of the fixation, intraocular lenses can either be anterior or posterior chamber lenses [70,73]. Modern cataract surgery is best when the fixated IOL is inside the capsular bag but if it is not damaged after the surgery, IOL can be positioned at a different location. As a result, each distinct site of fixation requires a different lens design that must be adjusted for implantation [70]. 

Silicone has been used also for tube shunts to reduce intraocular pressure (IOP) in glaucoma patients. Tube-shunt has a tube made of silicone that is placed in the eye to drain the fluid away from the eye, with several designs used in clinical applications [15,103,104].

Optimization of silicone chemistry, such as adding phenyl groups to the silicone polymer backbone, can affect transmittance and the refractive index (the ratio between the speed of light in a vacuum and the speed of light in a material). However, not all light is refracted by the material surface; it can also be reflected. Hence, if the material refracts more light than it reflects, it will appear more transparent. Since silicones are very transparent, this means that more light waves pass through the material than are reflected. The ability to adjust the refractive index value of silicone for different vision restoration requirements makes it a very suitable material for ocular implants [62]. Silicone is used for soft contact lenses that can also serve as a drug-eluting implant related to the anterior eye segment [9]. Improvements have been made to contact lenses by incorporating polydimethylsiloxane (PDMS) and tris-(trimethyl-silyl-propyl-methacrylate) (TRIS) to provide higher oxygen permeability, more flexibility, and reduced deposition of proteins by forming a hydrophilic soft gel layer which, altogether, result in a decrease in ocular infections and enable longer time to wear them during the day [105,106,107]. New contact lens solutions provide better integrity, improved tear resistance, and enhanced comfort [48,108].

One of the most significant material properties of IOLs is the water contact angle that reflects the ability of water to penetrate and diffuse through the IOL, which can provoke problems with blurred vision if water microdroplets are formed [109,110,111], and also has further influence on the posterior capsule opacification [112].

However, it should be noted that silicone oil should not be used in combination with silicone IOLs, due to the proven complications of oil attachment to the IOL surface resulting in optical irregularities [113].

#### 3.4.1. Silicone Intraocular Lens Effect on Posterior Capsule Opacification

The posterior capsule opacification, anterior capsule opacification, and glistening development after cataract surgery are all significantly influenced by the IOL’s manufacturing material, edge design, and optic [22]. The most typical post-cataract surgery consequence is posterior capsular opacification (PCO), which results in haze and blurred vision. PCO is typically treated using neodymium:YAG laser capsulotomy (Nd:YAG) or with chemicals that stop cell division [71]. The role of intraocular materials has been the subject of numerous research and regardless of the material, all IOLs are somewhat susceptible to PCO [69]. Studies have shown that the hydrophobic sticky surface of the IOLs enables its secure adherence to the capsule, inhibiting the growth of its epithelial cells, thus resulting in a reduction in the posterior capsule. Also, a sharp-edged design on hydrophobic IOL is better because it can prevent the migration of LEC to the IOL [109]. To give an updated assessment of long-term problems following the implantation of hydrophobic silicone and acrylic IOLs, Kwon et al. [21] reviewed and analyzed 10 studies involving 1138 eyes; IOLs were then compared and evaluated. There was no significant variance between silicone and acrylic IOLs when the follow-up time was excluded. Hydrophobic silicone IOLs with rounded edges, in contrast to acrylic IOLs with sharp edges, showed lower PCO values after six years of prolonged usage (the forming of Soemmerings ring abraded the sharp edges of acrylic IOLs over a long time). It can be concluded that rather than the edge design, the material properties have the biggest impact on the PCO value. In addition, silicone can resist the formation of the Soemmerings ring and mediate adhesion between the IOL and capsule through the combination of collagen IV and vitronectin adhesion proteins. Therefore, the hydrophobic silicone IOL can help prevent PCO for longer during long-term use [21]. However, Perez-Vives [22] showed that the edge design of the biomaterial is the most important factor in the prevention of PCO and lower Nd-YAG, considering silicone IOLs and hydrophobic acrylics with sharp edges as the best materials to be used. Adhesion of EMC components in vivo, such as collagen and fibronectin to the surface of IOL, may enhance cellular adhesion and contribute to opacification [69]. Silicone intraocular lenses exhibit very low levels of cell adhesion [74]. Wang et al. [114] investigated the adhesion, migration, morphology, and epithelial–mesenchymal transition (EMT) of human HLEB3 cells. In previous studies, silicone IOLs are known for cell low adhesion, whereas the same was confirmed in this study. The findings of this study indicated that silicone IOLs had low levels of cell adhesion on their surface that was comparable to PMMA IOLs but still greater than hydrophilic IOLs. While silicone IOLs also showed lower EMT values than PMMA, there was not a significant difference in the proportion of EMT between silicone and other IOL groups. It is significant to note that the IOL surface also influences the level of cell adhesion (roughness, smoothness, and irregularities).

#### 3.4.2. Silicone Orbital Implants

More than seven decades ago, the first silicone-based implant was produced for the repair of the bile duct [115]. Nowadays, surgeons prefer the use of porous silicone orbital implants and studies have shown that they give less postoperative complications such as orbital infection and inflammatory reactions [11,12]. Xu et al. created porous silicon scaffolds with excellent mechanical properties and porosity [116]. In vitro and in vivo tests showed that these scaffolds are good for both adhesion and proliferation and are not cytotoxic. They are easy to manipulate and support fibrovascularization, which is favorable for orbital reconstruction, thus making them a good candidate for orbital implants [116]. Porous implants effectively support fibrous ingrowth and increase the stability of the implant and its better attachment including positioning of the possible peg system [11]. The fabrication process is challenging. Some new methods involving 3D digital reconstruction have been studied to design and fabricate customized prostheses whose related costs are affordable [117].

#### 3.4.3. Glaucoma Drainage Devices

Insufficient drainage of aqueous humor causes glaucoma, an eye disease that causes a pathological increase in intraocular pressure that, if unregulated, can lead to blindness. In cases where the therapy fails or is not applicable, glaucoma drainage implants are used to drain the excess aqueous humor and lower the intraocular pressure [118]. An increase in the intraocular pressure can also be observed after vitreoretinal surgery [119]. Commonly used glaucoma drainage devices are the valved Ahmed, non-valved Baerveldt implant, and the original Molteno implant [13,14]. The Ahmed valve glaucoma drainage device has a complex mechanism and several parts all made of silicone [104]. Silicon tube placement can be realized in different ways, including the cases when IOL is present [14,103]. Liu et al. demonstrated that glaucoma drainage devices effectively stabilized the visual field [120].

## 4. Emerging Uses for Advanced Ophthalmology Treatments

Many ophthalmological conditions, especially chronic ones requiring prolonged treatment over a longer time, do not have fully adequate solutions and some eye conditions are still not curable. New types of drugs and new methods of drug administering, together with new material designs and the improvement of existing ones, are studied to provide new treatments. For example, emerging nanotechnologies promise to contribute to more efficient solutions [121,122,123,124,125] and especially to drug delivery systems [33,126,127]. Conventional therapy for ocular disorders cannot deliver an absolute cure because of the blood–retinal barrier’s limits in the eye when medications are applied [128]. Because the ocular barrier limits the bioavailability of given medications to just 5%, maintaining an effective drug concentration at the site of action for a fair amount of time is a significant challenge [128,129]. The ocular surface with its three-layer tear film, the corneal epithelium with its tight junctions and desmosomes, and the blood–water barrier of the ciliary epithelium, which is not pigmented, are the parts of the eye that obstruct the passage of molecules from the blood to the interior of the eye, whereas the blood–retinal barrier, made up of the pigment epithelium of the retina and the endothelium of the retinal arteries, prevents the passage of molecules from the blood into the retina and vitreous cavity. Blinking and tear drainage via the tear duct also contribute to minimizing the residence time of topically applied drugs [129]. Nanomedicine studies nanoparticles in clinical treatment, diagnosis, and management of diseases [129,130]. Since the 1980s, drug delivery systems as a treatment for many ophthalmological diseases have been studied [121,129], including bioadhesive enhancement, sustainable release, stealth function, stimuli–responsive release, and specifically targeted delivery. The structure of nanocarriers has advanced and these novel ocular drug-delivery systems include nanoparticles, nanomicelles, ocular inserts, liposomes, nanoemulsions, nanosuspensions, dendrimers, nanotubes, fullerenes, quantum dots, ferrofluids, and nanoparticle-loaded contact lenses [129,130]. The biocompatibility and potential cytotoxicity of the nanoparticles should also be considered. There is a chance that nanoparticles (NPs) will build up on the surface of the eye, which could disrupt cellular metabolism and impede tissue function, and possible inflammatory and allergic reactions [129].

### 4.1. Silicone Contact Lenses Loaded with Nanoparticles

Therapeutic soft contact lenses are increasingly used for drug delivery because they can reduce the side effects of drug-wasting eye drop therapy and have been shown to increase drug bioavailability by more than 50% [32,129,131]. Nanoparticle-loaded contact lenses can be made by incorporating various drug-coated particles, such as polymeric and inorganic nanoparticles, microemulsions, micelles, and liposomes [132]. The nanoparticle-loaded lens releases the drug into the tear film between the air and the lens and into the tear film between the cornea and the lens over an extended period of time. In this way, these lenses provide controlled drug release over 5 to 10 days compared to 2–5 min for drugs delivered via eye drops [129]. The type of contact lenses used most frequently are silicone-based hydrogel lenses, which can have a significant impact on both the corneal physiology and the medication loading and release characteristics. Since they can absorb and hold fluids and swell in a water-solvent platform, hydrogels are excellent for sustained ocular drug delivery systems. They hold solvents with other hydrophobic and hydrophilic agents, small compounds, and macromolecules [133].

Glaucoma is the second most common chronic ocular disease in the world, defined by an increase in intraocular pressure, which can lead to optic nerve degeneration and loss of vision [90,134,135]. Medications such as timolol maleate, β-adrenergic blockers, and miotics such as pilocarpine are most often applied as the treatment [135]. Direct drug loading and drug-packaging solution methods have disadvantages such as changes in the swelling and optical properties of the lens, low absorption of the drug, and excessive loss of the drug during direct filling of the lens [134]. 

Aiming to increase the penetration of ophthalmic drugs into the cornea, Mehta et al. [136] used electrohydrodynamic atomization (EHDA) to produce drug-loaded polymer fibers for silicone hydrogel lenses, which contain permeation enhancers. Permeation enhancers serve to increase drug absorption and ocular bioavailability. The research included different permeation enhancers and their effects on the in vitro behavior of the coatings made by electrospinning on the silicone contact lenses filled with timolol maleate. poly(vinylpyrrolidone) (PVP), poly(N-isopropylacrylamide) (PNIPAM) fibers containing permeability enhancers and loaded with timolol maleate were synthesized by electrospinning technology (EHDA) and evaluated using different thermal, in vitro, and spectral models. Borneol, a natural essential oil, proved to be the best permeation enhancer for prolonged drug release as 20 percent more drug was released compared to nanofibers without permeation enhancers. To determine the biocompatibilty of these formulations, freshly extracted bovine corneas were used.

Prostaglandin analogs, such as travoprost, latanoprost, and bimatoprost, are also used in glaucoma treatment. Moreover, timolol maleate [135,137] showed that, with the use of gold nanoparticles, it is possible to improve the uptake and release of bimatoprost for glaucoma treatment. The evaluation of the gold nanoparticles (GNPs) revealed that their average size was 21.1 nm; thus, they do not affect silicone lens optical properties. Bimatoprost, which is easily diffused on the surface of the GNP silicone lens, was better absorbed by contacts with immobilized gold nanoparticles inside the lens matrix. The large hydrophobic surface of the gold nanoparticles allowed for easy drug absorption, making them appropriate for drug release over a longer period of time. The in vitro test revealed a generally good rate of drug release, without a substantial amount of drug being released suddenly in the first hour and an extended-release lasting up to 72 h. The in vivo test demonstrated the potential of GNP lenses for prolonged release of the medication in the eye by demonstrating good biocompatibility of GNP lenses with the eyes of the tested rabbits and the presence of a high concentration of bimatoprost in the rabbit’s tear fluid at all tested time points. However, there is an issue with the burst release of the drug in the initial hours that might produce hyperemia; the low rate of release of the medication after 48 h may be below the therapeutic concentration, which should be addressed in later research. Investigations into the long-term toxicity of gold nanoparticles are also important (longer than 3 weeks). 

Another common eye disease is microbial keratitis [138], which is most often encountered by people who wear contact lenses. Microbial keratitis, as the corneal tissue infection, can lead to permanent vision loss due to corneal damage and perforation, if not treated. It is caused by pathogenic microorganisms such as *Staphylococcus aureus*, *Pseudomonas aeruginosa*, and *Serratia marcescens*, which are found on the surface of the contact lens and, during infection, form a biofilm that is quite resistant to disinfectants [135,139]. There are >18 bacterial strains that can influence reactions from different sources [140] and it is important to design a lens material to prevent possible infections. Metallic nanoparticles are also widely used in ophthalmology for treating microbial keratitis due to their high antimicrobial properties. To test the toxicity, antibacterial activity, and physicomechanical characteristics of silver nanoparticles in vitro, Mourad et al. [141] added them to silicone hydrogel contact lenses. The findings demonstrated that AgNPs exhibit remarkable antibacterial action, do not induce toxicity, and do not alter the silicone lens’ optical properties. In contrast, a review on the safety of silver nanoparticles published by Xu et al. [142] revealed that these particles can be potentially harmful. According to one study, silver nanoparticles can be hazardous to human eyes during early development and cause a variety of eye abnormalities. AgNPs’ potential cytotoxicity is influenced by their size, shape, application method, and concentration [129]. Despite their extensive therapeutic use, greater focus needs to be placed on researching the issue of their possible cytotoxicity on cells, tissues, and organs. Hence, a natural alternative to silver nanoparticles should be considered. For this reason, Sahadan et al. [139] created a functional polymer matrix made of polyvinyl alcohol that functions as a nano-scale drug delivery system for silicone contact lenses. Nanoparticles of phomopsidione, measuring 77.45 nm, were enclosed in PVA. This study examined the impact of phomopsidione nanoparticles coated on silicone hydrogel contact lenses in order to cure microbial keratitis. Phomopsidione (C7H1OO4) is a novel ketone derivative derived from *Diaporthe flaxinii* ED2. It has been demonstrated to be lowly toxic. The use of phomopsidione nanoparticles resulted in the complete inhibition of the growth of certain bacteria, thus indicating that the drug can be effective in the treatment of microbial keratitis. In this instance, the medication was released in a controlled manner, with 17 percent of the drug being released within 48 h. The medicine is released slowly and continuously in the first phase but rapidly and with a high concentration in the second phase since the silicone hydrogel has completely swelled. Tests should be conducted in the future to determine whether phomopsidione has antifungal capabilities.

### 4.2. Surface Modifications of Silicone Intraocular Lenses

Surface modifications of lenses, especially IOLs, can provide short- or long-term treatments by incorporating a drug-eluting function into the lenses [127]. Modified surfaces can provide medication transport for different treatments but can also increase biocompatibility [74,109]. Recent research in surface modifications of IOLs showed possibilities to mitigate and avoid adverse effects after surgeries [143,144]. Such modifications have considered spin coating, grafting, spray coating, and nanoparticle attachment, aiming to prevent cell adhesion and growth [144,145]. One of the most recent methods involves filling the IOL haptic with a slow-release system that contains a variety of antibiotics, anti-inflammatory medications, and anti-proliferation pharmaceuticals [26,144]. However, clinical trials are still needed. Due to their ability to reduce inflammation and limit the adhesion, migration, and proliferation of lens epithelial cells, modified intraocular lenses have been shown in numerous studies to reduce PCO [146]. A more hydrophobic surface improves capsular biocompatibility whereas a more hydrophilic surface improves uveal biocompatibility.

Mehta et al. [147] conducted a study to examine the effects of adding borneol and chitosan to polymer coatings on silicone lenses that were loaded with hydrogel and thymol maleate after it was demonstrated that borneol is an excellent potential permeability enhancer. Most ophthalmic drugs are hydrophobic and therefore cannot penetrate all layers of the cornea, so the use of permeation enhancers successfully bypasses this ocular barrier. Chitosan is a polymer with good properties such as low toxicity and mucoadhesiveness and is used to enhance permeability and further improve bioavailability to the eye [32]. To create stable and effective nanocoatings on silicone lenses, two polymers poly(N-isopropylacrylamide) and poly(vinylpyrrolidone) were electrically atomized and employed for drug encapsulation. The findings demonstrated that chitosan’s swelling capacity has a noticeable impact on the release of timolol maleate, increasing it by 23% compared to composite TM coatings and 11% compared to borneol-filled nanocoatings. The nanomatrices were discovered to have excellent intraocular biocompatibility and adequate size and to be very stable. This study is the first of its type to show how EHDA can be used to modify polymer formulations for drug release, potentially creating a new area of study for the field of ocular drug delivery.

Lin et al. [23] created a multilayer of chitosan and hyaluronic acid on the silicone surface to increase biocompatibility. The study’s conclusions showed that this HA/CHI-coated silicone IOL is efficient at preventing PCO because lens epithelial cells adhere less and proliferate less both in vitro and in vivo. In order to demonstrate the antibacterial power of HA/CHI multilayer, Lin et al. [148] modified a hydrophobic silicone intraocular lens surface by coating it with this soft and hydrated natural multilayer polysaccharide and polyelectrolyte multilayer (PEM) for treating endophthalmitis. Postoperative endophthalmitis is one of the most serious cataract surgery complications, where bacteria adhere to the IOL surface, proliferate, and form a biofilm. The most common pathogenic microbes are the Gram-positive micrococcus *Staphylococcus epidermidis* and *Staphylococcus aureus*. Because of the good hydrophilicity of PEM and the antibacterial activity of CHI, bacterial adherence was reduced and successfully destroyed in vitro. In vivo, implantation of this surface-modified IOL has also revealed less intraocular inflammation. 

One of the methods used for surface modification is achieved from polymerization reactions by obtaining polymer brushes [149,150]. Reversible Addition Fragmentation chain-Transfer (RAFT) polymerization has been employed increasingly frequently in biomedical applications due to its lower toxicity and lack of need for metal catalysts [24]. In the study of Lin et al. [24], PEG brushes were modified on silicone IOLs by surface-initiated RAFT (SI-RAFT) polymerization to improve biocompatibility. Due to the hydrophilic nature of PEG brushes, in vivo and in vitro testing demonstrated acceptable biocompatibility; the PCO was suppressed (Soemmering ring formation was significantly reduced) and the adherence of proteins, bacteria, and LEC was severely inhibited. Han [151], Wang [152], and Junmei [153] all applied polymer brushes to the silicone intraocular lens surface in the same manner. Silicone IOL was coated with poly(carboxybetaine methacrylate), a superhydrophilic zwitterionic polymer, by surface-initiated RAFT polymerization to improve IOL biocompatibility and reduce PCO, as shown by Han et al. [151]. The silicone material’s hydrophilicity was increased with excellent biocompatibility. In order to reduce postoperative endophthalmitis, Wang et al. [152] synthesized a (methacrylisobutyl polyhedral oligomeric silsesquioxane-co-2-(dimethylamino)-ethyl methacrylate) (p (MA POSS-co-DMAEMA)) brush on a silicone intraocular lens, which is more smooth and more hydrophilic than PDMS. In order to gain bactericidal function, the (p (MA POSS-co-DMAEMA)) brushes were quaternized by 1-bromo-heptane. The results showed a significant reduction in epithelial cells and bovine serum albumin adherence thanks to the hydrophilicity and cytotoxicity of the brush surface. Also, the bactericidal properties of brushes were very effective against *Staphylococcus aureus*, making it clear that this modified PDMS IOL can reduce the occurrence of PCO after cataract surgery. This study showed that materials modified on IOL surfaces can also have antibacterial effects. In a similar manner to Lin et al. [24], Junmei et al. [152] coated a PEG brush on a silicone surface. Results from in vitro tests indicated that this hydrophilic PEG brush-coated silicone has a lot of potential for implantation because they demonstrated decreased protein adsorption on the surface and a clear resistance to lens epithelial cells and *S. aureus* adhesion. Xu et al. [154] showed that another PEGylation modification of the silicone IOL surface can minimize the formation of PCO. By using chemical grafting with plasma assistance, hydrophilic polyethylene glycol (PEG) was put to the surface of silicone lenses. The silicone IOL’s optical characteristics were unaffected by PEGylation and in vitro tests revealed decreased LEC adherence on the PEGylated silicone IOL surface. The modified silicone IOL underwent in vivo implantation in rabbits and the outcomes demonstrated good in vivo biocompatibility, indicating that this hydrophilic modification can significantly lessen posterior capsular opacification. 

Based on a previous study by Lin et al. [136,147], which showed how well modifying HA/CHI silicone IOL improves biocompatibility, Huang et al. [25] modified a silicone IOL in the same way by also adding an antiproliferative drug to it for sustained drug release. Layer-to-layer fabrication was used to create HA and CHI on the intraocular lens’ surface. In this study, paclitaxel (Pac) was incorporated into HA chemically and employed as an antiproliferative medication. The HA-Pac/CHI multilayer demonstrated good hydrophilicity and an efficient anti-proliferation effect and in vitro drug release has demonstrated that the multilayer is stable under physiological conditions and has good sustained drug release. These findings suggest that the HA-Pac/CHI multilayer modified silicone IOL offers a novel approach to lower the incidence of PCO.

In their other study, Huang et al. [155] showed that specific chemical reactions that produce reactive oxygen species (ROS) can effectively assist in better IOLs, including drug delivery, such as using the enzymes horseradish peroxidase (HRP) and glucose oxidase (GOD) to cause cell apoptosis [121]. On the surface of silicone IOL, GOD and HRP were immobilized using mesoporous silica nanoparticles (MSNs) to construct the cascaded catalytic platform (enzymes@MSNs) [31,155]. Due to their favorable physiochemical characteristics, such as pore volume, size, structure, particle size, and surface functionality, MSNs are excellent nanocarriers that can hold and release a variety of medicines and biomolecules [156]. In vitro, results of enzymes@MSNs-IOL showed significant PCO reduction and high cell apoptosis, while in vivo testing demonstrated great intraocular biocompatibility to surrounding ocular tissues. The reason for this is that enzymes@MSNs-IOL have effective catalytic therapies. For example, the GOD catalyzes the conversion of intraocular glucose into gluconic acid and H_2_O_2_ and the HRP catalyzes the conversion of H_2_O_2_ into more toxic OH, which causes a high rate of LEC apoptosis. Moreover, the adjusted IOL’s optical qualities have remained excellent. Because it uses cascade catalysis for effective PCO prevention rather than toxic drugs, this innovative technique has a promising future in clinical settings [155].

#### Influence of Modifications on the Optical Properties of IOLs

It is essential that the optical characteristics of an IOL filled with drugs remain unchanged. Lamprogiannis et al. [26] used a spin-coating method to form a drug-eluting thin film on a silicone substrate to deliver dexamethasone (DXM) from the intraocular lens. The films were made of one- and two-layered thin films based on organic polymers [poly(d,l-lactide-*co*-glycolide) (0.65:0.35 w), poly(d,l-lactide-*co*-glycolide) (0.75:0.25 w), and polycaprolactone], and dexamethasone. Before sustained and persistent drug release took hold, drug burst occurred in the single layer of PLGA-PCL films in the first few hours, which had a higher ratio of drug encapsulation. Dexamethasone release was tested over a 10-week period and the findings indicated that the polymer film would be suitable for use in intraocular drug delivery systems due to adequate drug encapsulation and correct release, while IOL transparency and drug release rates were acceptable. It was deposited on a square silicone substrate, which might not be suitable for IOL surface modification; therefore, more research is required.

To determine if loading of the steroid dexamethasone has an effect on IOL optical properties (modulation transfer function (MTF), spectral transmission, and diopter power), Artigas et al. [157] conducted a second investigation with a hydrogel (pHEMA)-silicone intraocular lens. MTF, a measurement of the lens’s optical performance, significantly decreases following drug loading. However, the IOL’s optical quality is restored when the drug is administered; thus, a patient might need to wait a few days until the drug is released in order to recover their vision. The loaded dexamethasone had no effect on the IOL’s diopter power and it had a minor negative impact on the spectral transmission, which, like with MTF, recovered once the medication was released. Studies reviewed in this section are summarized in Table 2.

Even with all the benefits of advanced technologies and novel solutions, namely intraocular lenses, their clinical applications are rather far away since clinical trials are still missing [145]. Modification of the lens surface can provide additional benefits and improvements related to the antibacterial effect as well as reduction in inflammation and proliferation of LECa, which is also of great clinical importance [144,145]. The high cost of experimental research and clinical trials in these areas both present obstacles to more rapid application, as well as the need for more studies to comprehensively understand complex interactions of the influential factors determining the in vivo performance [158,159].

### 4.3. Exploring Novel Applications of Silicone in Ophthalmology

Current research related to silicone-based materials in ophthalmological applications is related to different soft implants that can restore eye function, treat some conditions, or realize diagnostics and control through advanced novel biosensors. 

The foldable capsular vitreous body (FCVB) implant was designed to treat severe retinal detachment, proliferative vitreoretinopathy, or silicone oil-dependent eyes [85,160]. FCVB is positioned in the vitreous cavity after the pars plana vitrectomy and scleral incision, whereas silicone oil is administered to support the retina. Three-armed silicone capsule is used in the case of highly myopic eyes with foveoschisis as a novel technique for macular buckling with good results in anatomic and visual improvement [161]. Contact lenses based on silicone were studied to encapsulate an atropine implant to be used for a continuous drug-eluting function for the treatment of myopia and vision correction and their efficiency was validated in animal models [162]. Silicone-based lenses made to perform integrated functions of sensing and actuation showed excellent properties in the aspects of flexibility, strain, speed, and stability over time [43].

Silicone haptic stoppers were used for the fixation of intrascleral IOL and iris reconstruction, with a modified Yamane technique [163]. Another approach entails a self-healing hydrogel as an implant for a vitreous body that also provides tamponade to stabiles the retina and uses an alginate-based composite; in vivo results showed inhibition of retinal detachment recurrence [164].

Improvement in glaucoma valves has been studied by developing a dual-hydrophilic and antifouling coating on silicone [165]. Results from in vitro tests showed that such a coating can efficiently suppress and protect from protein contamination, oxidation, inflammation, and fiber proliferation. Results from in vivo tests showed that the coating significantly decreased the level of encapsulated fibers through the inhibition of inflammation and fibrosis.

The development of smart materials and biosensors opened up new avenues for ophthalmological sensors as well. For example, one of the common conditions that needs constant monitoring and frequent check-ups is high intraocular pressure (IOP). A combination of flexible polydimethylsiloxane (PDMS) with Fiber Bragg Grating (FBG) was studied to create a smart contact lens that can continuously monitor IOP [166]. The control of IOP changes in glaucoma patients can be realized by using an implant made of laser-responsive shape memory polymer and clinical lasers, as validated through in vitro trials [167]. Another silicone implant was developed with composite coating and the ability to release brimonidine for IOP treatment and in vitro, results were promising from the aspect of long-term IOP lowering [168].

Smart contact lenses can identify biomarkers in tear fluid and monitor biological parameters, including intraocular pressure and blood glucose [169]. Some prototypes that monitor glucose levels and intraocular pressure for up to 24 h have been developed but still need further improvement [170,171].

A bionic eye for patients with sight loss has been a subject of research for many years but the only approved implants for clinical use are still retinal implants, although transplantation of cornea or cataract surgery are commonly used. Some of the novel solutions currently investigated are 3D electronic implants in subretinal space (photovoltaic subretinal prosthesis) [172], microelectrode arrays for subretinal stimulation [173], neuronal signal recordings [174], or three-layer design of a structure closely resembling retina structures [175]. However, artificial eye implants that could provide full eye function are still out of reach with only a few existing solutions that can provide some partial eye functions. Nevertheless, even these retinal implants are valuable for patients who would otherwise suffer from complete vision loss. For example, second-generation retinal prosthesis can enable functional vision for patients suffering from end-stage retinitis pigmentosa [176]. 

Functional biomaterials in oculoplastic and orbital surgery represent the latest advancement of clinical biomaterials [27], together with the development of materials and devices for implantable bioelectronics [28]. These reviews of novel biomaterials and implants, including those for the reconstruction of the orbital floor with fractures, anophthalmic sockets, and retinal implants, pointed out some new research directions but also stressed that clinical trials are lacking related to the new material solutions. The development of fully functional smart biomimetic biomaterials is challenging in many aspects, such as with regard to the long-term safety of materials and the interfaces between the biomaterial and tissue or material structures that can enable encapsulation but the possible benefits fully justify further research. 

## 5. Challenges in the Development of Silicone-Based Materials for Ophthalmological Applications

Even though silicone has excellent properties, some issues still exist. Silicone intraocular lenses are slippery when wet [17,177], which makes it more difficult to manipulate them, and they also show very high adherence to silicone oil after vitreoretinal surgery. Therefore, silicone IOLs should not be implanted in highly myopic eyes at risk of retinal detachment [177]. Also, there are reported problems with the opening of silicone IOL in the anterior chamber after implantation. This can cause the rupture of the posterior chamber capsule [10,16,62,177]. They have a lower refractive index than acrylic IOLS, which makes them thicker; however, silicone hydrogel IOLs can be carbazole-grafted in order to generate a higher refractive index [71,177]. Also, newer versions of silicone foldable IOLs are continuously being developed, all in the direction of better short- and long-term postoperative results [68]. Some studies have also shown the potential risk of postoperative infections by using silicone IOLs due to the possible bacterial adhesion on silicone surface [10,17,18], such as the occurrence of opacification of silicone IOLs was present, with brown discoloration, due to different factors. Some of the reasons are pre-operative contamination (local spraying or insecticide agents) of semi-permeable packages of IOLs, anomalies in the manufacturing process, and impurities of silicone polymers, which can lead to opacification of the lens [18,178]. It is believed that the brown discoloration is from the water vapors that diffused in the silicone IOL [179]. Also, posterior surface calcification in silicone IOLs can occur in the presence of asteroid hyalosis [16,179]. The silicone IOL’s posterior surface has calcium deposits that can be removed with the Nd:YAG laser; however, the issue still exists since the deposits keep building up after the surgery. Asteroid hylasois is a degenerative illness in which calcium/phosphate bodies form and accumulate in the vitreous body and for silicone intraocular lenses, it can trigger unique opacification [70]. The advantages and disadvantages of silicone intraocular lenses are summarized in Table 3.

### Future Directions in the Development of Silicone-Based Materials

Future solutions in the development of silicone-based materials should provide better treatments, customized drug delivery, and enhanced patient comfort, including possible new cures for currently incurable ophthalmological conditions. 

Silicone modifications from aspects of microstructure and nanocomposites, including surface modifications [127], should further address friction coefficients in contact with the tissue and IOL injector to provide improvements in terms of the easier positioning of IOLs during the surgery to aid in overcoming its property of being slippery when wet [17,177]. Also, it would be beneficial to study how to mitigate or prevent adherence to silicone oil [177] and provide a non-stick contact mode during contact with the silicone surface. Different surface structuring, such as producing micro-brushes [149,150], has already shown promising effects related to silicone hydrophilicity and suppression of PCO conditions, also including decreased protein adsorption and bacterial adhesion [152]. 

Simultaneously, improvement in strength could be realized by further studying the reinforcement of silicone by different nanoparticles (such as Ag [137], Au [141], chitosan, and hyaluronic acid [23,136,147]) to address the possible opening of the silicone IOL in the anterior chamber after implantation [10,16,62,177] but also to provide anti-fouling and anti-bacterial properties, as they are currently recognized risks [10,17,18], and to prevent PCO [23]. It would be very beneficial to further develop silicone IOL materials that would, by their inherent properties, suppress the building and forming of calcium/phosphate in the vitreous body [70].

Further study of silicone foldable lenses will enable further development of foldable capsular vitreous body (FCVB) implants for the treatment of severe retinal detachment and visual improvements [161] but also to develop smart materials with biosensing functions to serve for simultaneous treatment, diagnostics, and control, by sensing, for example, high intraocular pressure and glucose and identifying biomarkers and to monitor other biological parameters [170,171]. 

Drug delivery systems have already shown exquisitely good results in treatments of different eye conditions [33,127,145] and their development will surely continue. The incorporation of materials that can trigger specific chemical reactions, like producing reactive oxygen species (ROS) [121,155], can enable novel advanced drug delivery systems and treatment for conditions that are currently incurable.

A very significant research area that should see new advancements in the future is the development of the bionic eye [172,173,174,175], which can be applied for many different purposes, including the treatment and cure for blindness, but also for further development of soft robots for use in various sectors. Research on the effects of electrical stimulation on tissues is needed, considering the fact that lab results related to functional biomaterials [27] and implantable bioelectronics [28,173] showed significant possibilities in treatment and tissue regeneration.

A comprehensive literature review, as presented in this paper, clearly showed significant advancements in ophthalmology from aspects of using silicone-based materials. However, further research is needed, especially including clinical trials in material development, which are the most important phase but are currently lacking, that will bring advanced solutions to patients, as the final research goal. 

## 6. Conclusions

This paper reviews the latest research and applications of silicone in ophthalmology, especially related to intraocular lenses (IOLs), but also with reference to other applications including contact lenses, scleral buckles, corneas, lacrimal ducts, glaucoma drainage systems, ocular endotamponades, and drug delivery systems. Silicone oil has been used as a standard material in eye surgery for long-term internal intraocular endotamponade, such as complicated retinal detachment repair. Intraocular lenses are permanent implants to replace the natural lens or for correcting vision by implanting it over the natural eye lens, whereas the first commercial foldable intraocular lens was made of silicone. 

Following cataract surgery, silicone intraocular lenses are placed in the eye to replace the natural lens and restore the patient’s normal vision. Intraocular lenses are, to some degree, susceptible to posterior capsular opacification (PCO), which is one of the most common complications that occur after cataract surgery. Biological aspects of posterior capsular opacification have been reviewed and biological reactions triggered by the surgical procedures, including the effects of the implanted silicone IOL and challenges in case of some medical conditions. Adsorption of the proteins on the IOL surface starts immediately after implantation and facilitates the adsorption of other cells. New approaches, like surface modifications, have shown possibilities to enhance silicone lens biocompatibility and reduce PCO but also to incorporate the drug-eluting function. 

Mechanical characterization of IOLs is shortly presented, including new approaches for in vivo testing. For a comprehensive understanding of IOL functionality and behavior, in vivo mechanical characterization would provide the necessary details but, currently, only a few technologies are in clinical use: mainly medical imaging technologies and spectroscopy. Even for in vitro and lab testing, only a few methods and standards exist, related to the mechanical properties of the IOLs’ material, whereas strain, creep, fatigue, and triboelectric properties should be determined in both in vitro and in vivo conditions. This is a challenge for the development of new advanced silicon-based biomaterials and new methods and measuring technologies should be further investigated and developed.

The latest trends in nanotechnology solutions for advanced ophthalmology treatments have been reviewed, especially related to drug delivery systems. Nanoparticle-loaded silicone contact lenses or IOLs are ideal candidates for drug delivery and prolonged eye treatments. Surface modifications of silicone intraocular lenses are reviewed and research showed that these can improve the surface of the lens in such a way that the lens provides long-term treatments for various medical conditions or medical diagnoses through incorporation of the sensory functions. Nanoparticles (NPs) used for drug delivery will not change the IOL optical characteristics due to their small size, as an obligatory request for drug-eluting ocular lenses.

The development of nanotechnology and nanomedicine has created innovative systems for the slow release of drugs from both intraocular and contact silicone lenses. It has been shown that the most effective way is by entrapping the drug in organic or inorganic nanoparticles and loading them on silicone contact lenses for sustained drug release. It would be a very good substitute for drugs in the form of eye drops, considering that the bioavailability of the drug from eye drops is reduced to only 5%. On the other side, drug-loaded silicone intraocular lenses could enable a slow-release system in the posterior parts of the eye. Despite their excellent therapeutic effect, biocompatibility of NPs should also be taken into consideration and their potential cytotoxicity should be further investigated. Nanoparticle-loaded drug-eluting lenses have demonstrated a very high potential for use as an innovative method in the treatment of the most prevalent and complex eye diseases. However, it is necessary to conduct preclinical and clinical studies to confirm whether their long-term application is safe in order to achieve their commercialization at all, which is a very challenging and time-consuming process.

## Figures and Tables

**Table 1:** Physicochemical properties and biocompatibility of silicone.

Physicochemical Properties	Biocompatibility
Hydrophobicity	Hemocompatibility
Significant flexibility	Less vulnerable to host tissue attacks
Low glass transition temperature	Repeated sterilizing does not harm silicones
Chemical inertness, bioinertness	Does not provoke genotoxicity or transformation of healthy cells in cancer cells
High elasticity	Suitable for long-term implantations
Good electrical properties	Antiadhesive characteristics
Optically transparent	Specific adhesive characteristics
Thermal oxidation resistance	

**Table 2:** Studies on surface modification and drug loading on silicone IOLs.

Drug Modification	Surface Modification	Technique	Study Aims and Results	References
	CHI/HI multilayer	Layer-by-layer	PCO reduction	Lin et al. [23]
	Ha/CHI and PEM multilayer	Layer-by-layer	Postoperative endophthalmitis	Lin et al. [148]
	PEG brushes	SI-RAFT polymerization	PCO reduction, antibacterial	Lin et al. [24]
	PMPC brush	SI-RAFT polymerization	PCO reduction	Han et al. [151]
	(p (MA POSS-co-DMAEMA))	SI-RAFT polymerization	Postoperative endophthalmitis	Wang et al. [152]
	PEG brushes	SI-RAFT polymerization	PCO reduction, antibacterial	Junmei et al. [152]
	PEG brushes	Plasma-assisted chemical grafting	PCO reduction	Xu et al. [154]
Antiproliferative drug Paclitaxel	HA/CHI multilayer	Layer-by-layer	PCO reduction	Huang et al. [25]
	HRP/GOD immobilized using MSNs	Layer-by-Layer	PCO reduction	Huang et al. [155]
Anti-inflammatory drug dexamethasone	PLGA-PCL film on silicone substrate	Spin coating	Testing optical changes in IOL	Lamporgianis et al. [26]
Dexamethasone	DMX incorporated into PHEMA-modified silicone IOL matrix	Thermo polymerization	Testing optical changes in IOL	Artigas et al. [157]

**Table 3:** Advantages and disadvantages of silicone intraocular lens.

Advantages	Disadvantages
Flexible and made to be implanted with a small incision	Slippery when wet
Adjustable refractive index value	High adherence to silicone oil
Hydrophobic IOL’s sticky surface enables good adherence to the capsule, inhibiting the growth of epithelial cells and resulting in a reduction in the posterior capsule	Opening of silicone IOL in the anterior chamber, potential risk of postoperative infections due to the possible bacterial adhesion on the silicone surface
PCO prevention for longer during long-term use	Brown discoloration
Low level of cell adhesion, resulting in good uveal biocompatibility	Posterior surface calcification in case of the asteroid hyalosis
